# First report of *Aedes albopictus* (Diptera: Culicidae) in the North of Colombia

**DOI:** 10.1590/S1678-9946202365049

**Published:** 2023-09-15

**Authors:** María Claudia Atencia-Pineda, Alfonso Calderón-Rangel, Richard Hoyos-López, Javier García-Leal, Rafael Bolaños, Paula Pareja-Loaiza, Ronald Maestre-Serrano

**Affiliations:** 1 Universidad de Córdoba, Doctorado en Microbiología y Salud Tropical, Montería, Córdoba, Colombia; 2 Universidad de Córdoba, Facultad de Medicina Veterinaria y Zootecnia, Instituto de Investigaciones Biológicas del Trópico, Montería, Córdoba, Colombia; 3 Universidad Simón Bolívar. Facultad de Ciencias de la Salud, Barranquilla, Atlántico, Colombia

**Keywords:** Aedes, Surveillance, Introduced species, Colombia

## Abstract

*Aedes albopictus* is considered a potential vector of arboviruses in Colombia. Females and males naturally infected with dengue, Zika and chikungunya viruses have already been found in this country. We document the first record of *Ae. albopictus* in the Cordoba department, in North of Colombia. The finding was carried out during *Ae. aegypti* collection activities in the Ayapel, Montelibano, Planeta Rica, Pueblo Nuevo and Puerto Libertador municipalities. The entomological material was collected in water containers such as cement water tanks, tanks, bottles, tires, abandoned toilets, and plastic lids with natural water located in the intradomicile, peridomicile, and extra-domicile spaces of the homes. We collected 658 *Ae. albopictus* samples in the larva and pupa stages, and once these reached adulthood, we determined that 389 were female and 269 were male. This is the first record of the presence of *Ae. albopictus* in the Cordoba department.

## INTRODUCTION

*Aedes albopictus* (Skuse, 1894) is a mosquito species originally from Southeast Asia^1^. In Asia, *Ae. albopictus* is also important for transmitting dengue virus, but in the Americas, it is a potential vector of dengue, Zika and chikungunya^2^. This species has ample adaptive capacity to natural and urban ecosystems due to its physiological characteristics such as diapause in eggs exposed to extreme temperatures and larvae that can occupy various types of natural breeding sites, like the axils of leaves, tree holes, bamboo, bromeliads, and artificial breeding sites like tires and water tanks^3^.

*Ae. albopictus* is a diurnal, exophagic species with different daily bite timeframes that depend on host availability, breeding habitats, and temporary climate regimes^3^. It uses different species as sources of blood meals (birds and mammals), but is predominantly anthropophilic^4^. This opportunism in its feeding range improves biological parameters associated with reproductive capacity, population colonization, occupation of ecological habitats in the woods–urban area interphase^5^, and the transmission of zoonotic pathogens from animals to humans, which highly impacts public health and is the reason why arboviruses constitute a special interest^6^. *Ae. albopictus* is also considered a public health problem due to its role in chikungunya and dengue epidemic outbreaks in Europe^7,8^, where there is a record of natural infection and vector competence of 24 arboviruses from the *Alphavirus*, *Flavivirus*, *Orthobunyavirus*, *Phlebovirus*, *Orbivirus*, and *Picornavirus* genera, and parasites like *Dirofilaria* spp., *Plasmodium lophurae*, and *Plasmodium gallinaceum* ^9^.

*Ae. albopictus* wide geographical distribution has been recorded in Africa, Oceania, Europe, and America. In the latter, it has been reported in various countries in both the continent and Caribbean islands, from the USA^9^ to Argentina^10^. In Colombia, *Ae. albopictus* was first recorded in 1998 in an abundantly vegetated suburban area of the Leticia municipality, located in the Amazonas department^11^, and since then, it has been recorded in 54 localities in 14 of the country’s departments, including the departments of Amazonas, Antioquia, Caldas, Casanare, Cauca, Choco, Narino, Putumayo, Quindio, Risaralda, Santander, Valle del Cauca, Arauca and Cundinamarca^10^. The quick population and geographical expansions associated with this species’ biological and ecological characteristics, along with the persistent transmission of the dengue virus, the introduction of chikungunya in 2014 and Zika in 2015 to Colombia, and the circulation of other emergent and reemergent arboviruses in various ecological zones, demand a bigger effort in the entomological surveillance of this species, which is considered a potential vector of arboviral diseases of great importance in public health. Regarding arboviruses such as dengue, Zika and chikungunya, the epidemiological situation of Cordoba department is critical, because it is considered a geographical zone with high dengue transmission, with approximately 29,249 dengue cases reported from 2010 to 2022. Since the beginning of the chikungunya epidemic, the period between 2014 and 2022 recorded approximately 16,882 chikungunya cases. For Zika, since its introduction in 2015, until 2022, there were approximately 4,108 cases^12^. However, the notification of chikungunya and Zika cases decreased one year after the epidemiological peaks, 2014 and 2015, respectively^12^.

The objective of this research is to document the first record of *Ae. albopictus* in North of Colombia, Cordoba department.

## MATERIALS AND METHODS

The collection of *Ae. albopictuss* in immature stages was done during *Ae. aegypti* collection activities in the urban areas of the municipalities of Ayapel (8.313839218, -75.1460486), Montelibano (7.973777245, -75.41681813), Planeta Rica (8.408200286, -75.58324108), Pueblo Nuevo (8.978398524, -75.79306809) and Puerto Libertador (7.888858911, -75.67176143), in the Cordoba department, located in North of Colombia. The sampling was carried out in different neighborhoods of the target municipalities targeted between the months of June and November 2022 (during field trips). The entomological material collection was done intradomicile (inside the house), peridomicile (area around the house up to a distance of 10 meters) and extra-domicile (area greater than 10 meters around the house), in water containers such as cement water tanks, tanks, bottles, tires, abandoned toilets, and plastic lids with natural water. Plastic pipettes and mesh strainers were used to collect the larvae and pupae. The *Ae. aegypti*, still in immature stages, were transported to the insectary in airtight plastic containers, where they were kept in controlled temperature, relative humidity, photoperiod and biosecurity conditions until reaching adulthood and were ready for taxonomical confirmation by means of diagnostic characteristic observation using the keys of Rueda^13^.

## RESULTS

**Figure 1:**
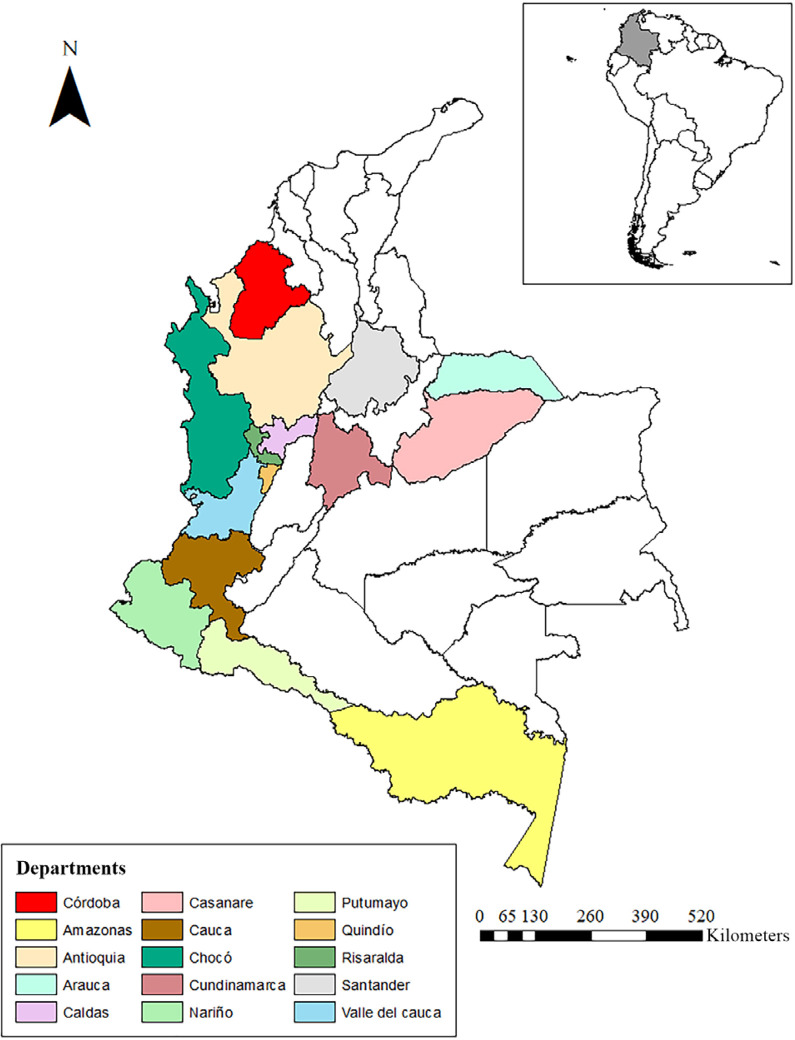
Geographical distribution of *Aedes albopictus* in Colombia, from the first report, in 1998, in the Amazon (yellow), to the most recent report, in 2022, in Cordoba (red).

**Figure 2:**
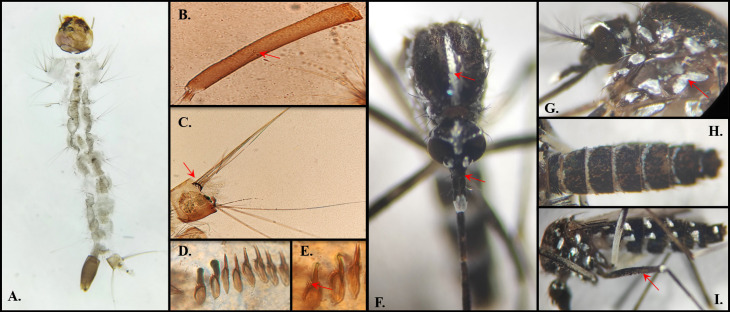
Diagnostic characters in *Ae. albopictus* larvae and adults: A) Dorsal view of the larvae; B) Antenna without spicules; C) Ventral brush (4-X) with four setae pairs; D) Comb spicules, regularly spaced; E) Comb spicules without subapical spines; F) Thorax: Scutellum with a medial-longitudinal white stripe. Head: Clypeus without white scale spots; G) Mesepimeron with non-separated white scales, forming a V-shaped white spot; H) Abdomen, abdominal tergum with complete basal white stripes; I) Anterior portion of the medial leg femur without a longitudinal white stripe.

Although the predominant species in the inspected hatcheries was *Ae. aegypti*, 658 *Ae. albopictus* adults were identified in the larvae collected (Table 1). From these, 103 were collected in Ayapel; 123 in Montelibano; 5 in Planeta Rica; 10 in Pueblo Nuevo and 417 in Puerto Libertador (Table 1). This finding means the geographical distribution of *Ae. albopictus* in Colombia is extended to 15 departments (Figure 1). The main diagnostic characteristics for larvae and adults were identified. The diagnostic characters observed to identify the larvae were serda VII with a double or triple head, thorax with short lateral spines; teeth of the segment VIII comb without subapical spines and, in adults, the clipping without spots of white scales, the thorax scutum with a narrow white median-longitudinal stripe, and median femur without longitudinal white stripe (Figure 2). The limitations of this study were that we could not characterize the breeding sites and the species’ abundance in the sample zone, keeping in mind that their finding was eventual, since the collection activities targeted *Ae. aegypti*.

**Table 1:** Characteristics of the municipalities where the collections were made, and total of identified *Ae. albopictus* males and females.

Municipalities	Months collection date	Population size (Urban population census 2018)	Altitude (m.a.s.l)	Climate		♂	♀	Total
				Temperature (°C)	Average monthly rainfall (mm)			
Ayapel	June	24,482	20	>28	200-300	37	66	103
Montelibano	November	55,906	50	26-28	150-200	50	73	123
Planeta Rica	August	40,411	67	26-28	200-300	1	4	5
Pueblo Nuevo	September	8,857	50	26-28	200-300	4	6	10
Puerto Libertador	November	11,670	52	26-28	150-200	177	240	417

m.a.s.l.= meters above sea level; mm = millimeters; °C = degrees Celsius. Population size data taken from the National Administrative Department of Statistics (DANE) and climatic data from the Institute of Hydrology, Meteorology and Environmental Studies (IDEAM), Colombia.

## DISCUSSION

Since the first record of *Ae. albopictus* in the Amazonas department, in southern Colombia, and up to date, its geographical dispersion route has been indicated by reports in the country’s western, central and eastern departments, which means this is the first record for this species in North of Colombia, Caribbean region. The accelerated distribution of *Ae. albopictus* around the world has been related to the commercial exchange of used tires^14^ and to the availability of ecological habitats as breeding sites^15^. Moreover, niche models have evidenced that dry wood remains immersed in the urban matrix are associated with this species’ presence and the availability of habitats under 3,000 meters above sea level^10^. Both conditions are possible in the Cordoba department, where dry wood remains, conservation areas, and swamp complexes exist; as well as appropriate ecological and climatic conditions for the survival of mosquito vectors^10^. The Ayapel municipality, for example, is located in a swamp complex, with climate variables (Temperature: 23-35 °C; Rainfall: 23-151mm; RH: 93-100%) that favor the *Aedes* species’ reproductive and survival cycles^16^. The route that enabled the introduction of *Ae. albopictus* to the Cordoba municipality is unclear; however, a possible one could have been through the Cauca river in the Antioquia department, since the species is related to this basin’s area of influence (the Valdivia and Ituango municipalities)^17^, which connects to the San Jorge River in the rural and urban areas of the Montelibano, Puerto Libertador, Ayapel, Pueblo Nuevo and Planeta Rica municipalities, through marshes and streams.

Subsequent molecular analyses could clarify the phylogeographical route or evolutive origin of this population, as observed in La Tebaida in Colombia’s Quindio department, where, by means of the COI gene – barcode, Singapore was identified as the probable origin of the identified insects and a constant genic flow was found with the population in Medellin in the Antioquia department^18^.

## CONCLUSION

To date, in Colombia, existing reports only demonstrate data on *Ae. albopictus*’ abundance, habitats, dispersion, and coexistence with *Aedes aegypti* ^10,15,19^; none of these reports addresses the hypothesis of *Aedes albopictus* ecological displacement by *Aedes aegypti*. According to what was observed in the study area, the dominant species in the inspected farms is *Ae. aegypti*, and the differences observed in our abundances of *Ae. albopictus* in relation to the municipalities are possibly associated with variations in the spatial-temporal arrangement of the hatcheries. Given the discovery of this species in the five municipalities, it is necessary to strengthen the entomological surveillance of *Ae. albopictus* in Cordoba and the other departments of the Caribbean region to determine its geographic expansion trajectory in the San Jorge and Cauca river basins and the ecological habitats in areas where it could coexist with *Ae. aegypti* in rural or peri-urban environments. The high dengue transmission in the study areas, the presence of the *Ae. albopictus* vector and the availability of breeding sites make entomological and virological surveillance necessary to clarify the vectorial role in this area of the Cordoba department.
